# Supplementary figures and images for: The evolution of separate sexes in waterhemp is associated with surprising chromosomal diversity and complexity
Source: PLoS Biol. 2025 Jun 30;23(6):e3003254. doi: 10.1371/journal.pbio.3003254 (PMC12237273; doi:10.1371/journal.pbio.3003254)

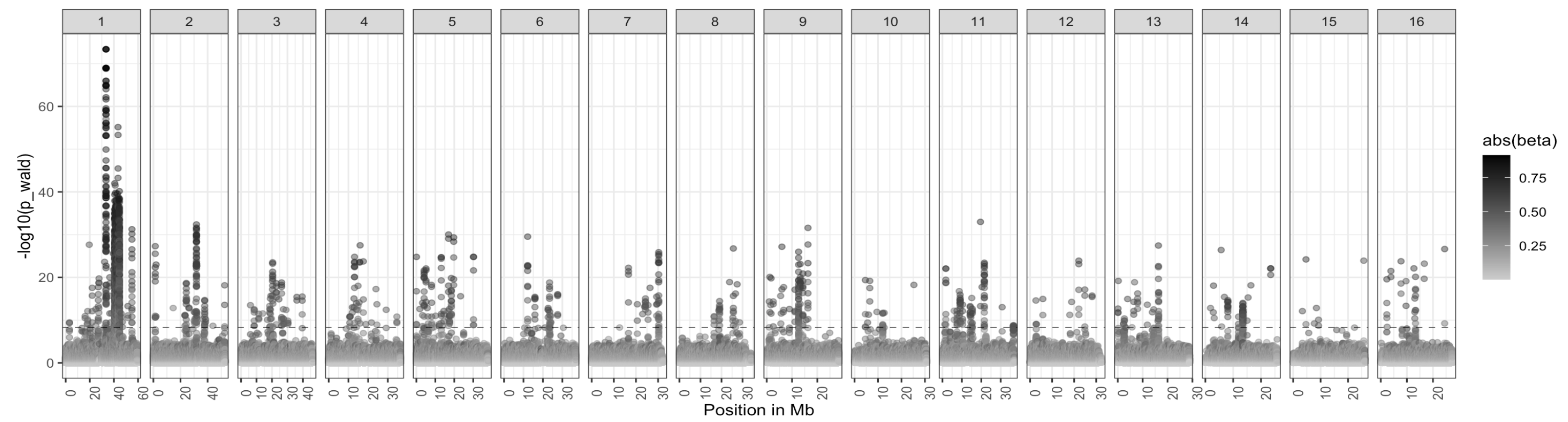

Supplement: S1 Fig — Analysis was done on SNPs called from reads mapped only to haplotype 2. The data underlying this figure can be found in https://zenodo.org/records/15594570. (PNG) [file pbio.3003254.s001.png]

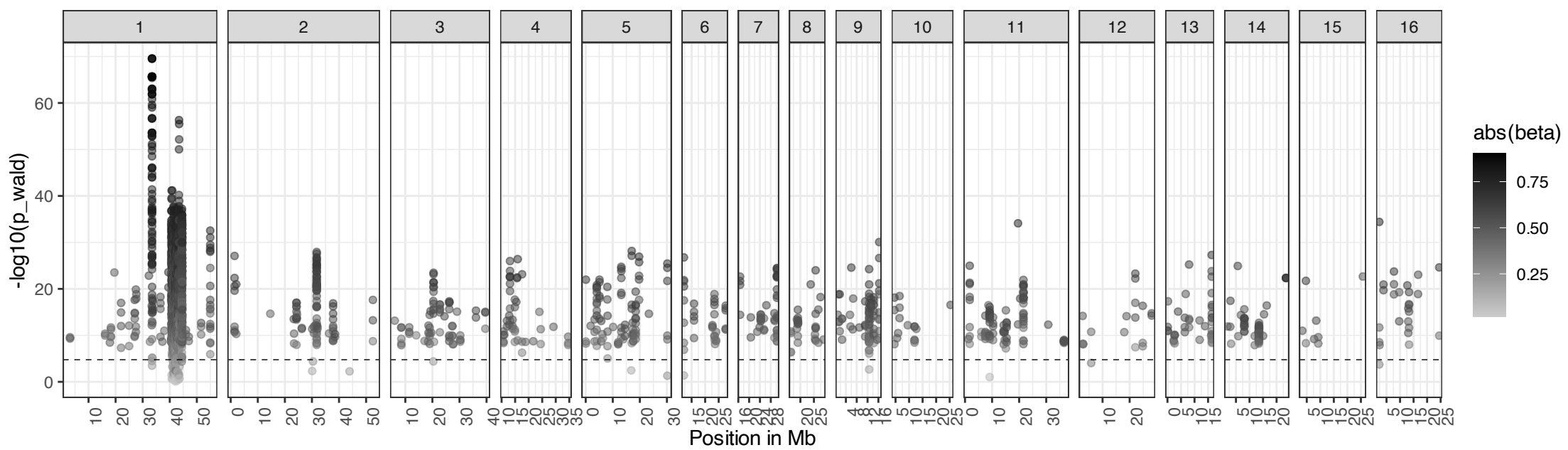

Supplement: S2 Fig — The data underlying this figure can be found in https://zenodo.org/records/15594570. (PDF) [file pbio.3003254.s002.pdf]

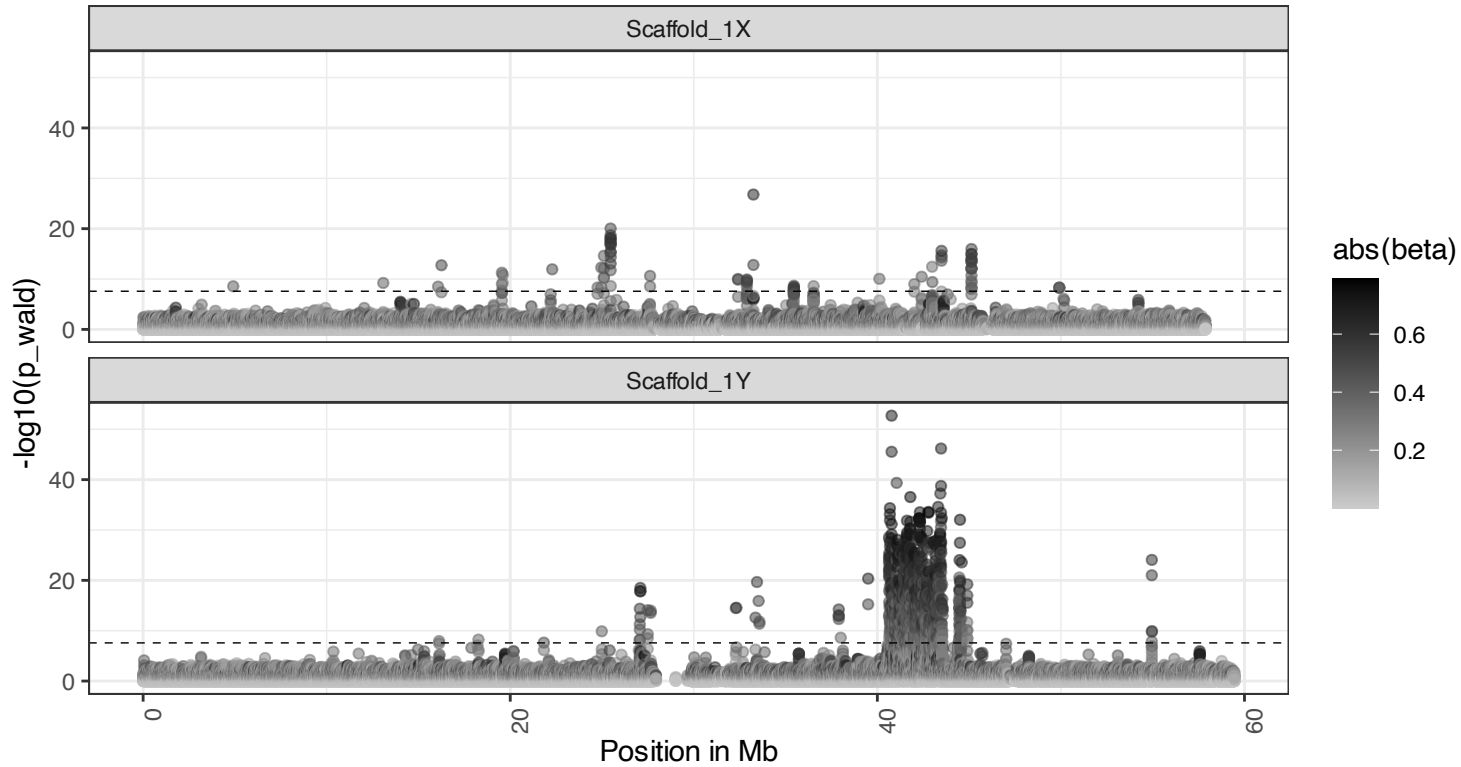

Supplement: S3 Fig — The data underlying this figure can be found in https://zenodo.org/records/15594570. (PDF) [file pbio.3003254.s003.pdf]

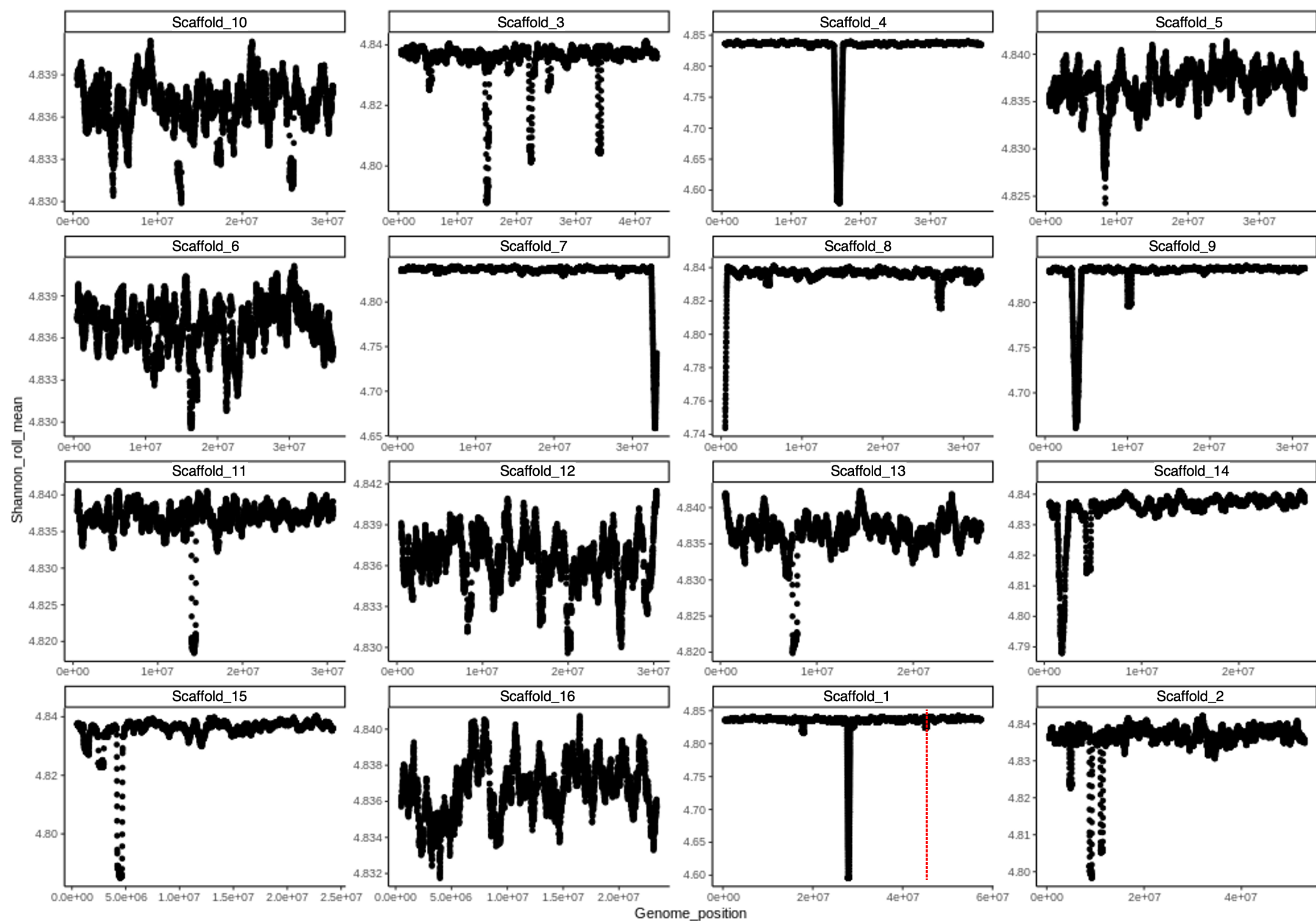

Supplement: S4 Fig — The sex-linked region is present on Chromosome 1 around ~41 Mb (vertical dashed red line), and shows a tertiary minimization of Shannon diversity. The data underlying this figure can be found in https://zenodo.org/records/15594570. (PDF) [file pbio.3003254.s004.pdf]

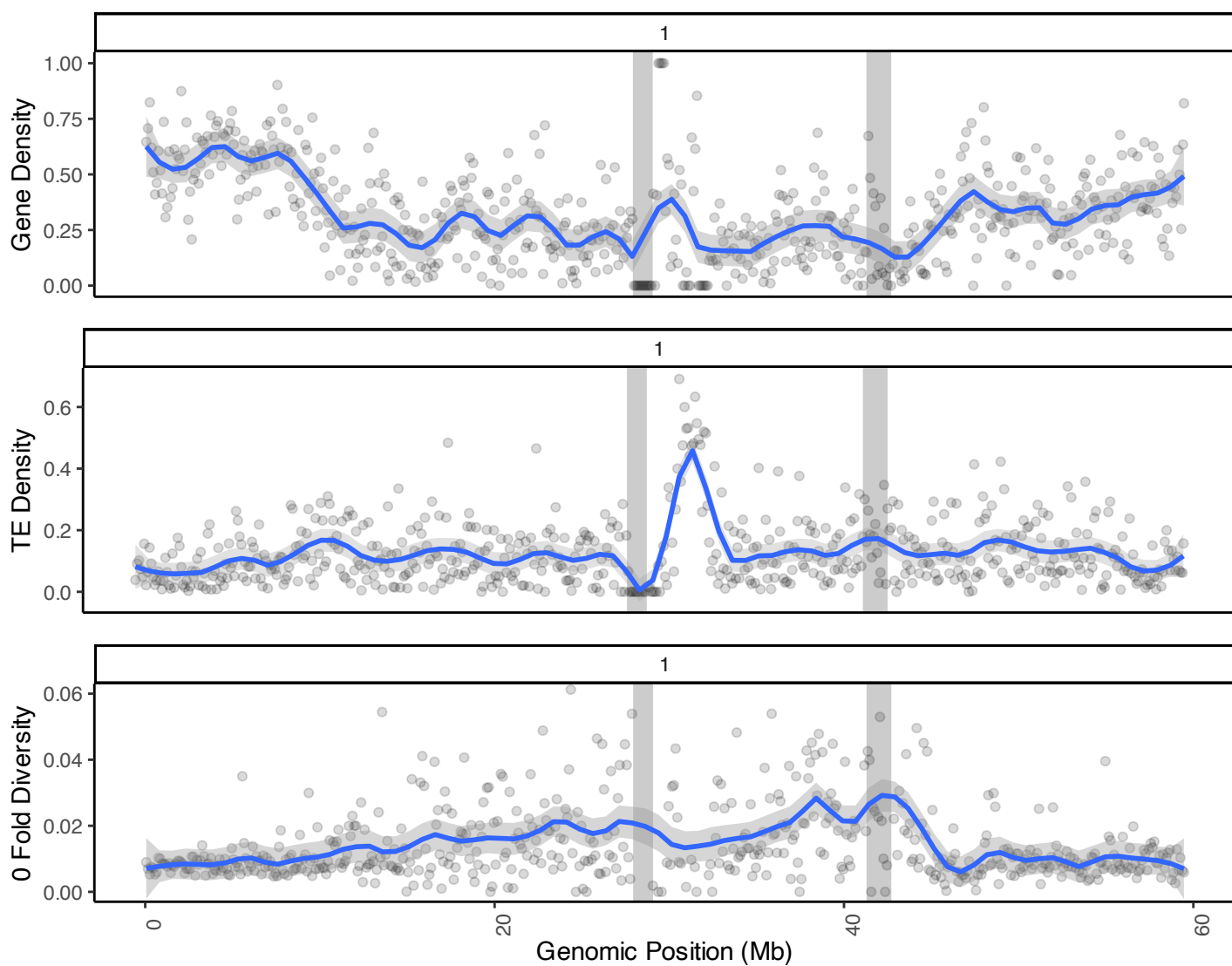

Supplement: S5 Fig — Transposon element (TE) density is greatly enriched at ~32 Mb, just neighboring the inferred location of the centromeric region (left vertical bar; as inferred from RepeatObserver). The data underlying this figure can be found in https://zenodo.org/records/15594570. (PDF) [file pbio.3003254.s005.pdf]

**A**

X Haplotype

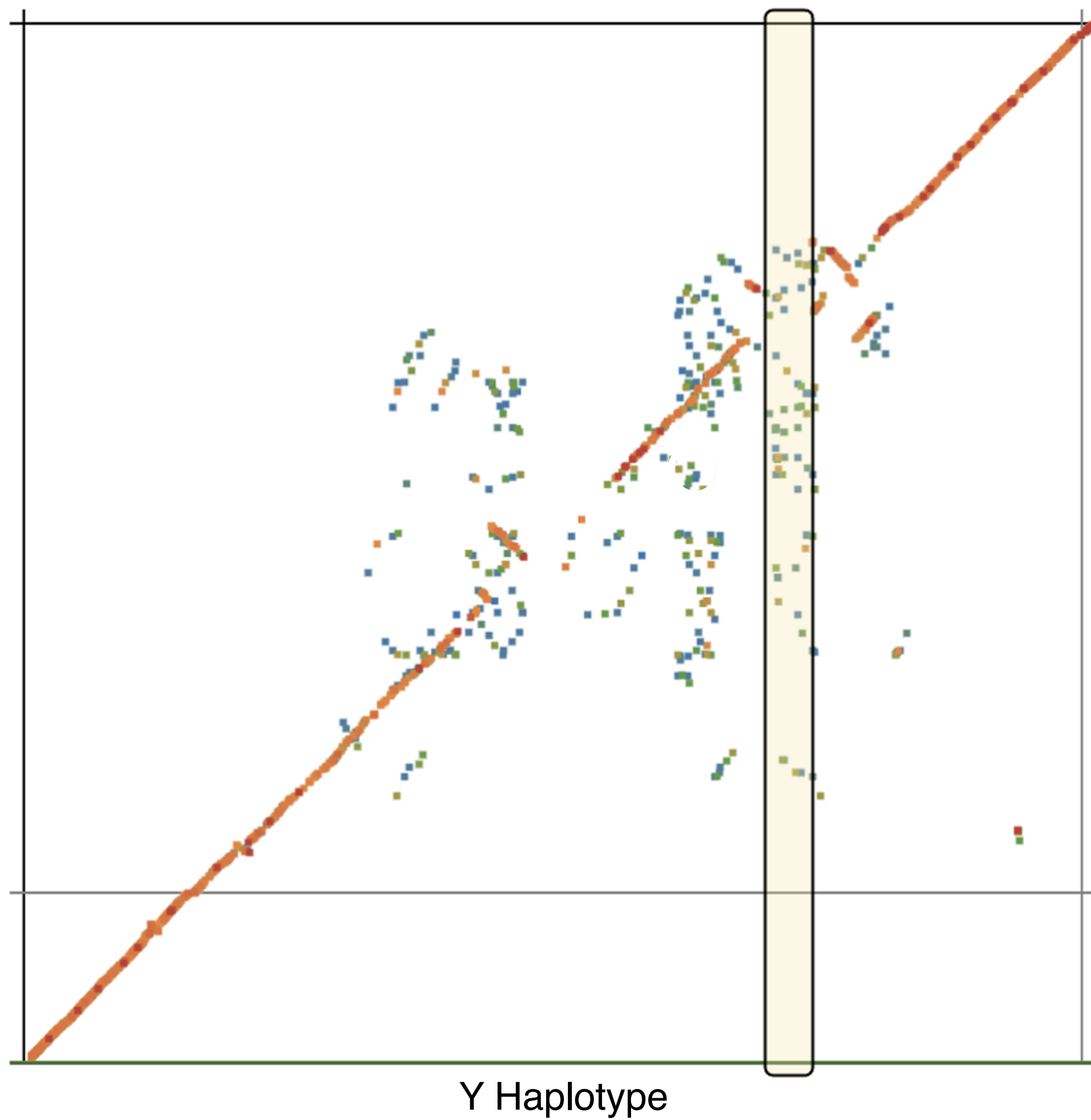**B**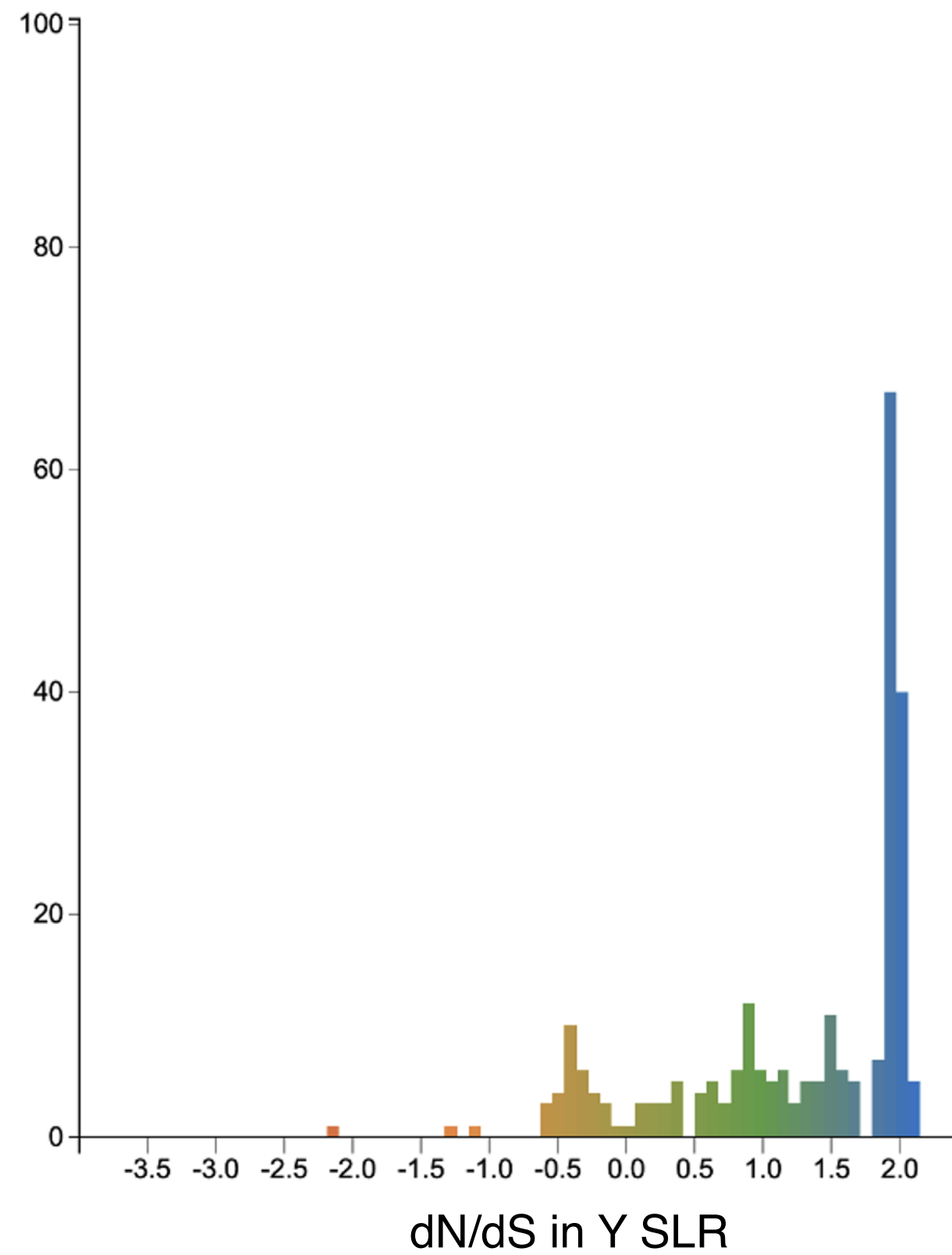

Supplement: S6 Fig — (A) Genic synteny analyses in CoGe [90] between the X haplotype and Y of our focal genome resolves the presence of duplicate genes across the X mapping to the Y. (B) Genes with synteny in this highlighted SLR show signatures of paralogy, with high levels of divergence (dN/dS). The data underlying this figure can be found in https://zenodo.org/records/15594570. (PDF) [file pbio.3003254.s006.pdf]

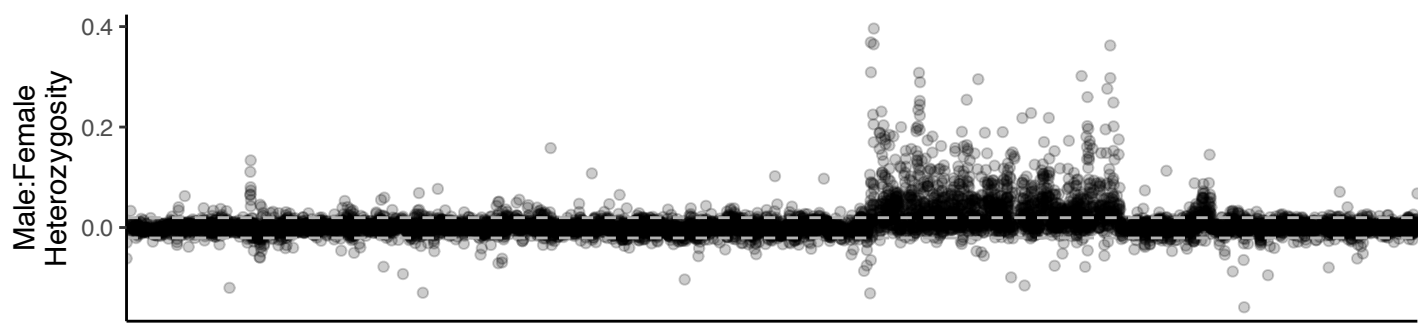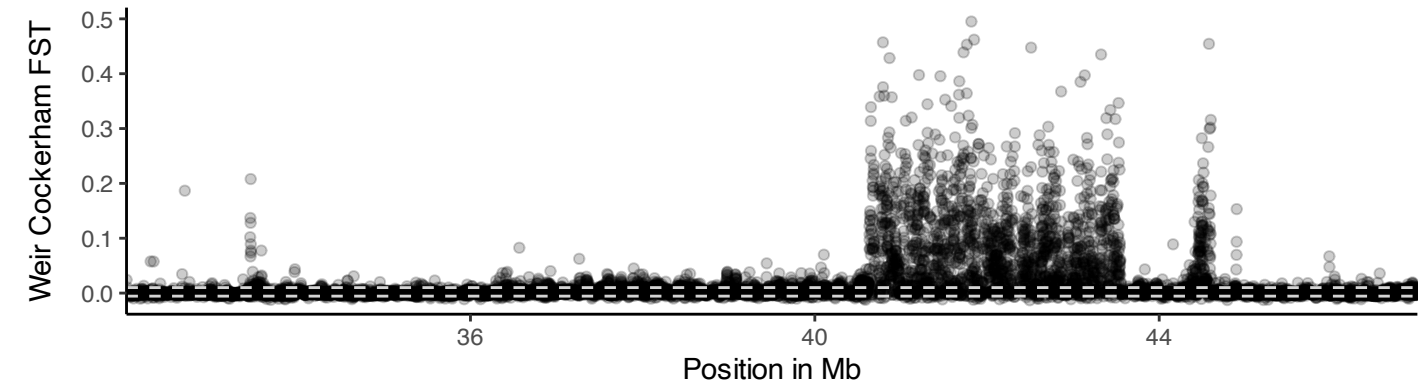

Supplement: S7 Fig — In both plots, the 95% percentile of these summary statistics across autosomes is delimited by the horizontal dashed white lines. Mean FST in the SLR [40.5–43.5 Mb] is 0.07, whereas the mean ratio of male:female heterozygosity is 0.037. The data underlying this figure can be found in https://zenodo.org/records/15594570. (PDF) [file pbio.3003254.s007.pdf]

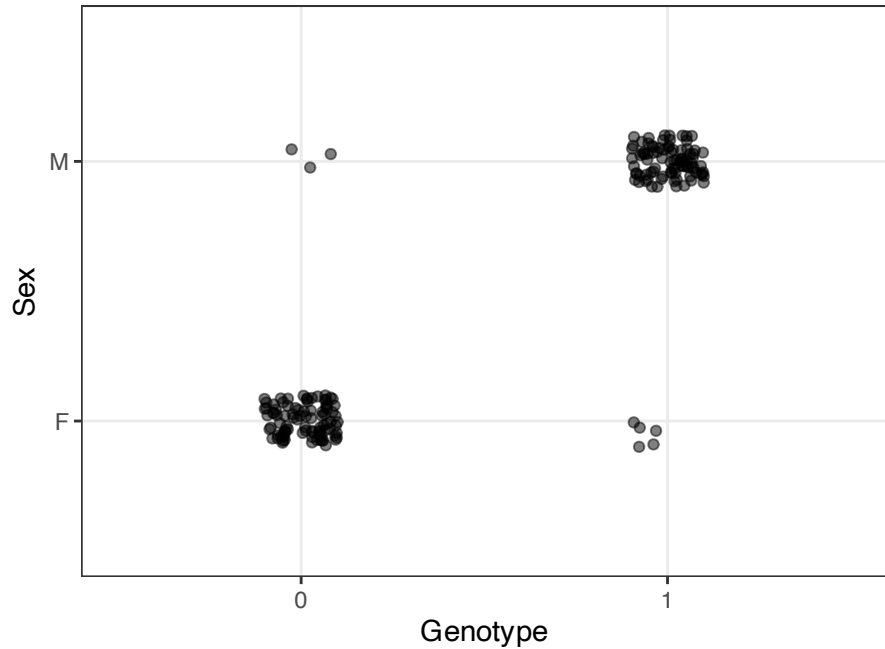

Supplement: S8 Fig — The data underlying this figure can be found in https://zenodo.org/records/15594570. (PDF) [file pbio.3003254.s008.pdf]

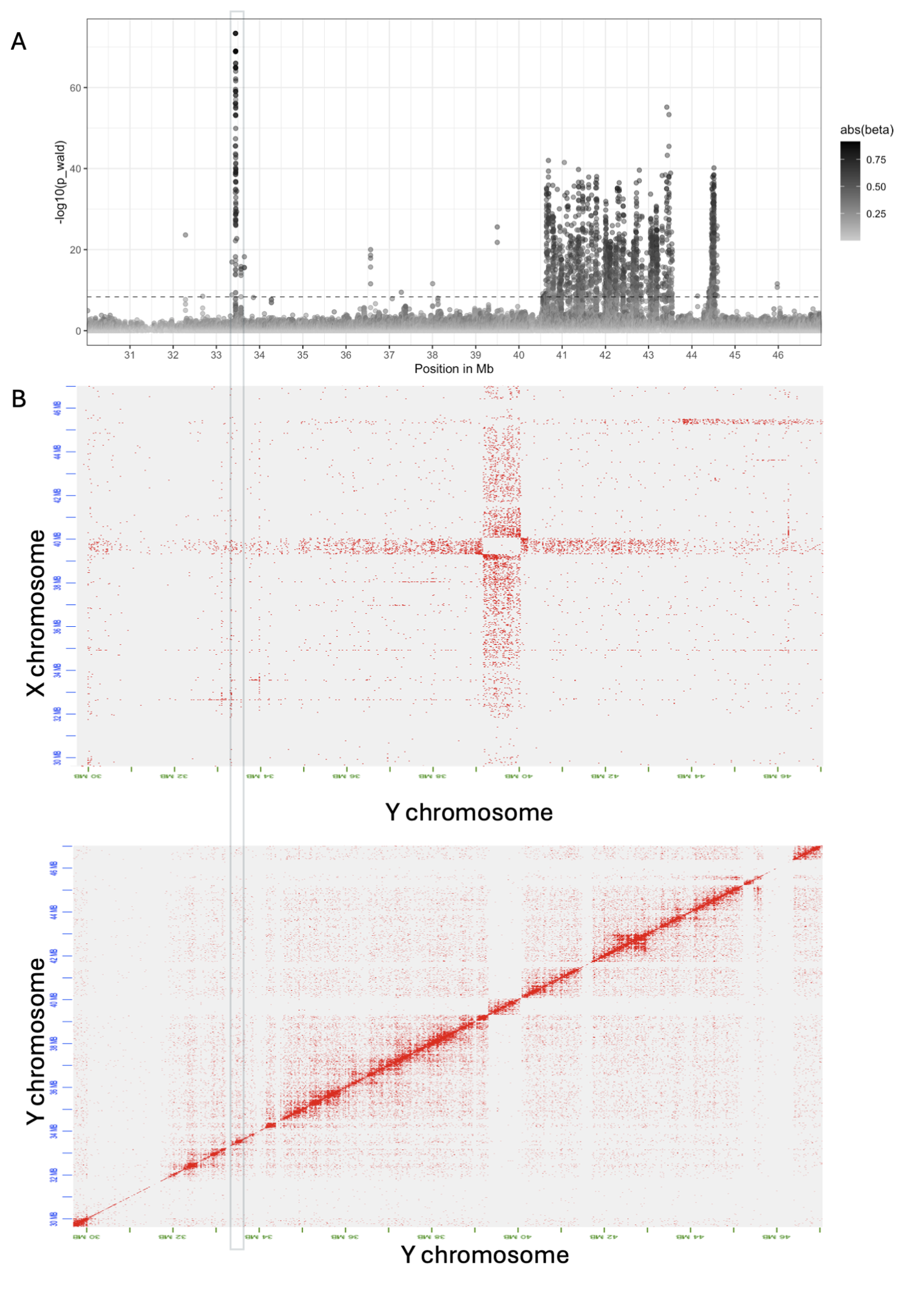

Supplement: S9 Fig — (A) A GWAS of SNPs on the sex-linked locus, highlighting an upstream peak at ~33.5 Mb. (B, upper) Hi-C contacts between the Y chromosome and the X chromosome support the correct phasing of the SLR, and despite an upstream region (not sex-linked) that appears to have been phase switched. (B, lower) Contacts within the Y chromosome (lower) support the placement of the highlighted sex-associated locus upstream [at ~33.5 Mb] of the primary SLR, and the correct phasing and placement of sex linked contigs within the SLR. The data underlying this figure can be found in https://zenodo.org/records/15594570. (PNG) [file pbio.3003254.s009.png]

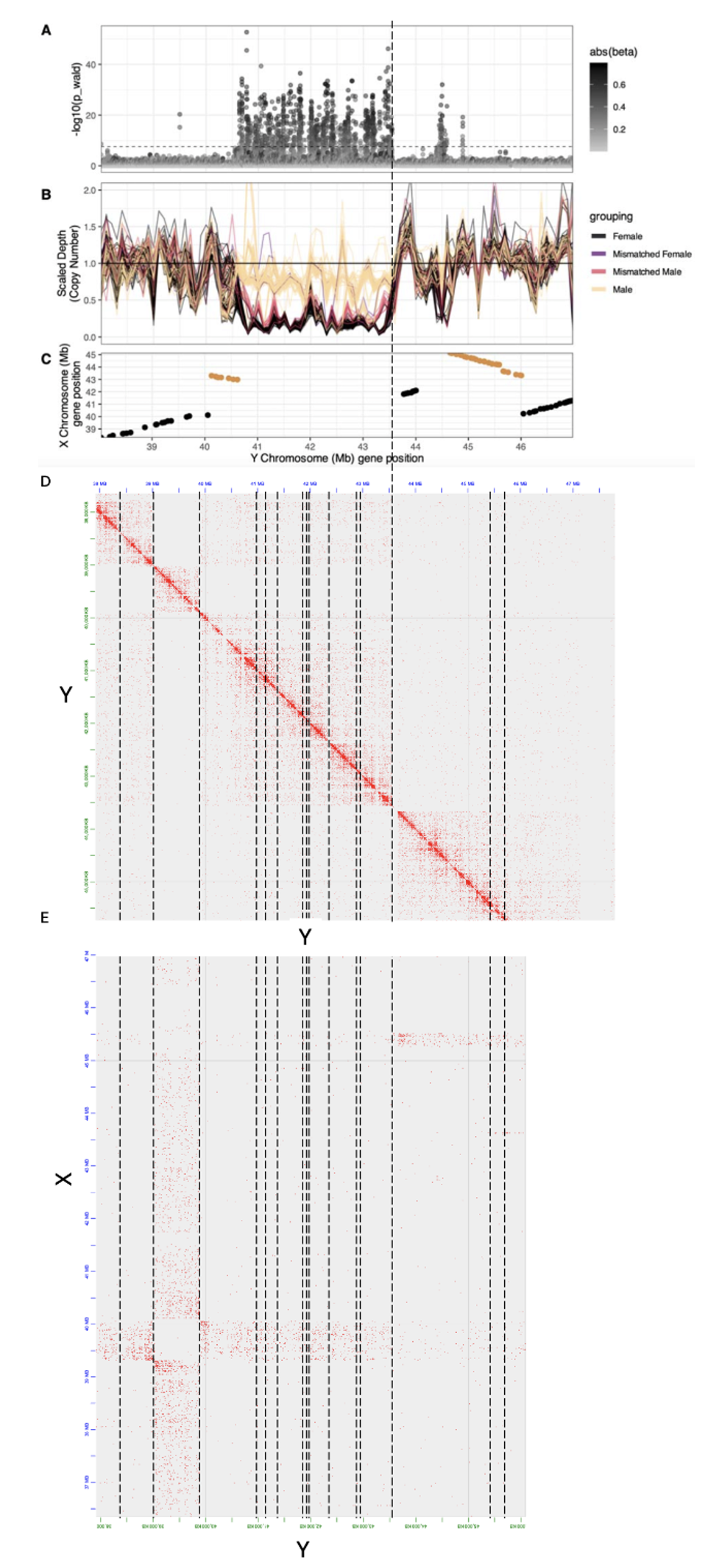

Supplement: S10 Fig — Evaluation of assembly quality near the sex-linked region (SLR) on chromosome 1 (A–C). Hi-C contacts within the Y chromosome (D) support the placement and phasing of the sex-associated peak downstream [at ~44.5 Mb] of the primary SLR. However, the right bound of the SLR does not have contacts with the downstream sequence, suggesting a large gap in our assembly. Contacts between the Y chromosome and the X chromosome (E) show a region with incorrect phasing (highlighted in red) that is not sex-linked. Gaps between scaffolded contigs in the assembly are indicated by vertical dashed lines. The data underlying this figure can be found in https://zenodo.org/records/15594570. (PNG) [file pbio.3003254.s010.png]

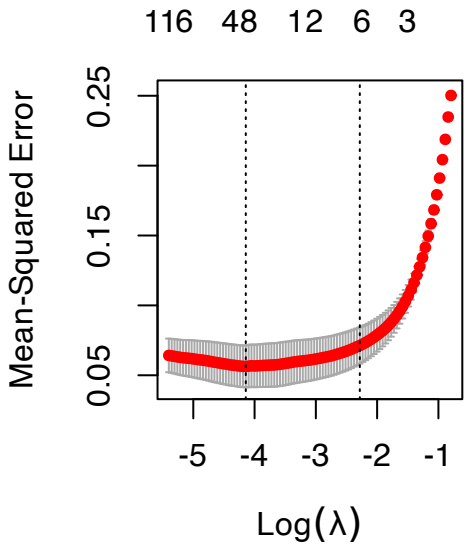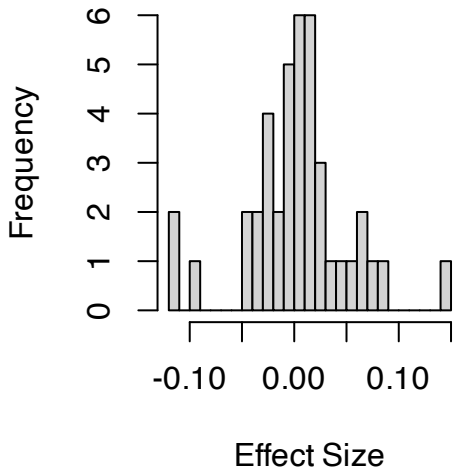

Supplement: S11 Fig — (Left) The minimization of MSE occurs when lambda = 0.016. (B) The distribution of effect sizes for the 42 loci with remaining non-zero effects on sex. The data underlying this figure can be found in https://zenodo.org/records/15594570. (PDF) [file pbio.3003254.s011.pdf]

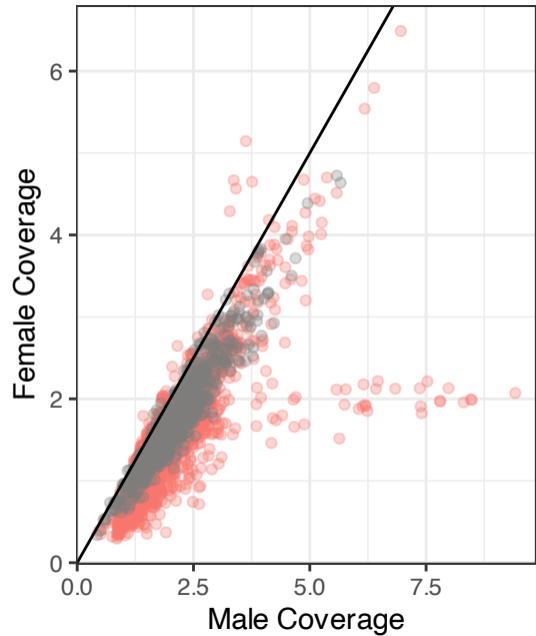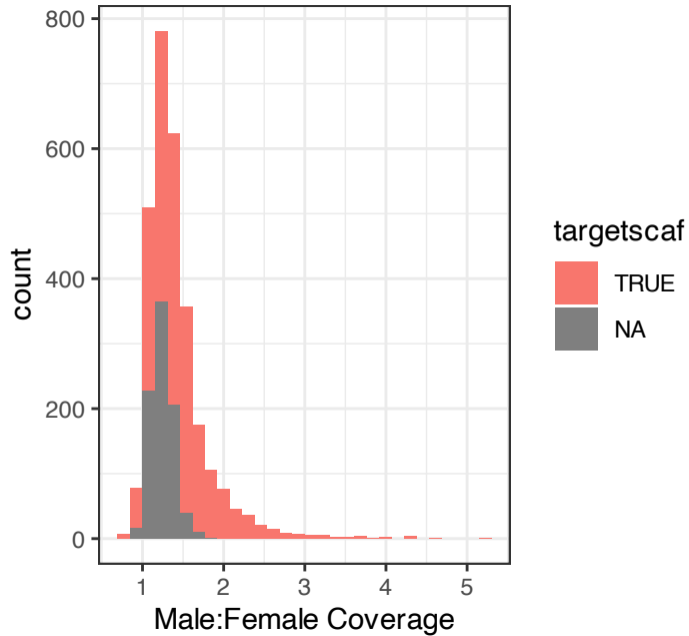

Supplement: S12 Fig — Color indicates whether loci are located on the sex-linked, “target” Chromosome 1, or on other scaffolds across the genome. The data underlying this figure can be found in https://zenodo.org/records/15594570. (PDF) [file pbio.3003254.s012.pdf]

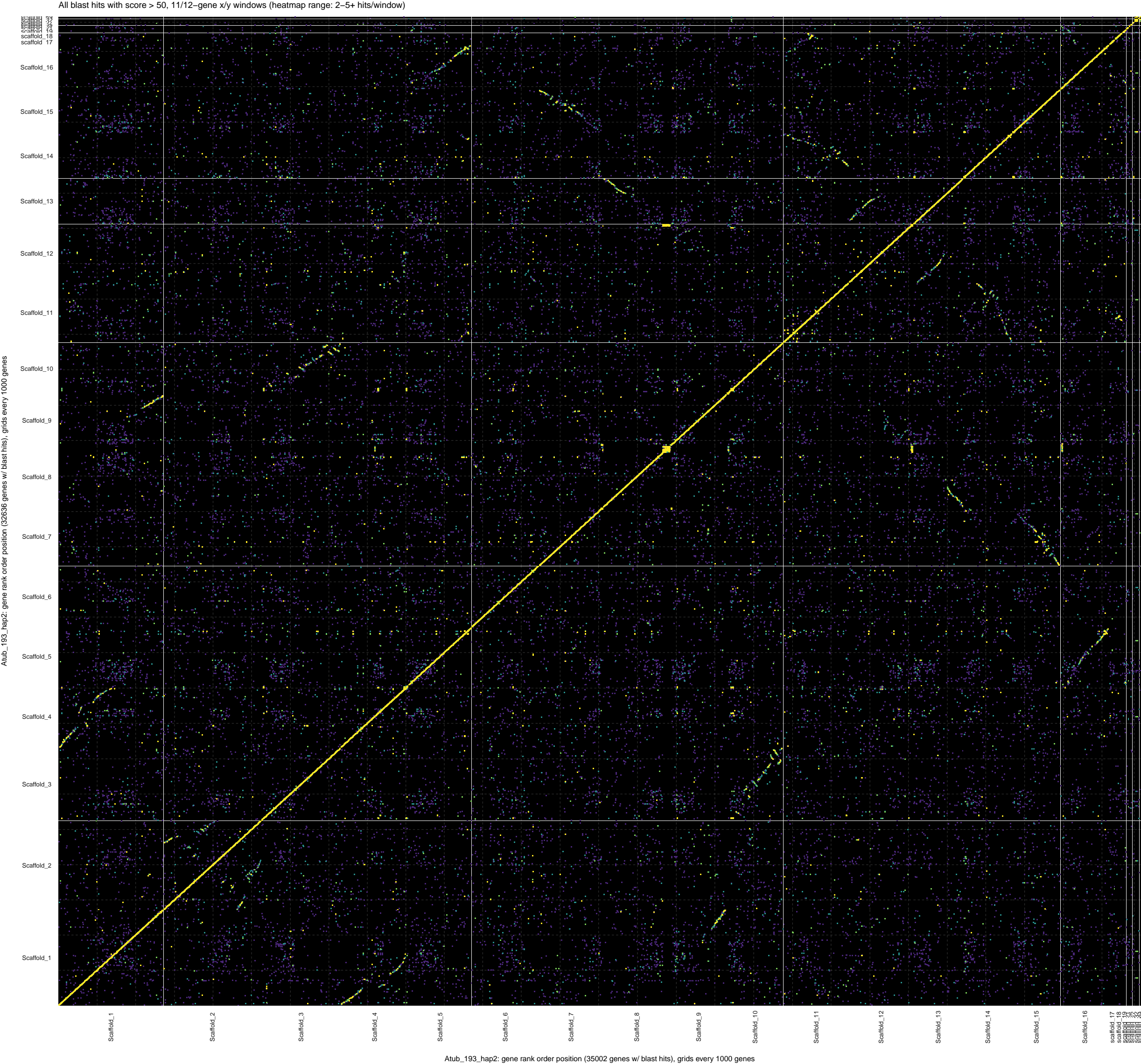

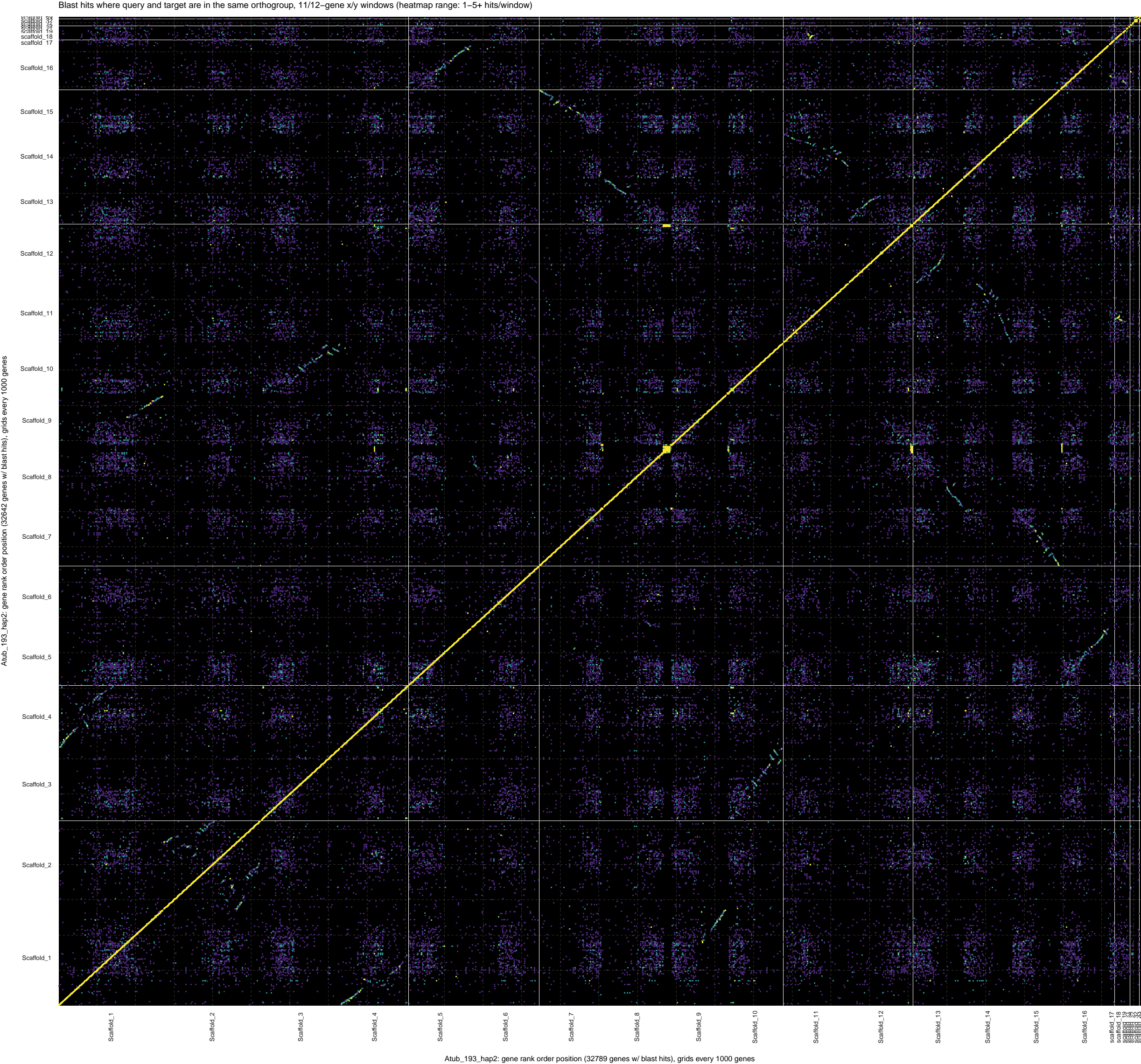

Supplement: S13 Fig — Evidence of a past whole-genome duplication event is apparent as every scaffold shows contiguous stretches of synteny with one or more scaffolds. The data underlying this figure can be found in https://zenodo.org/records/15594570. (PDF) [file pbio.3003254.s013.pdf]

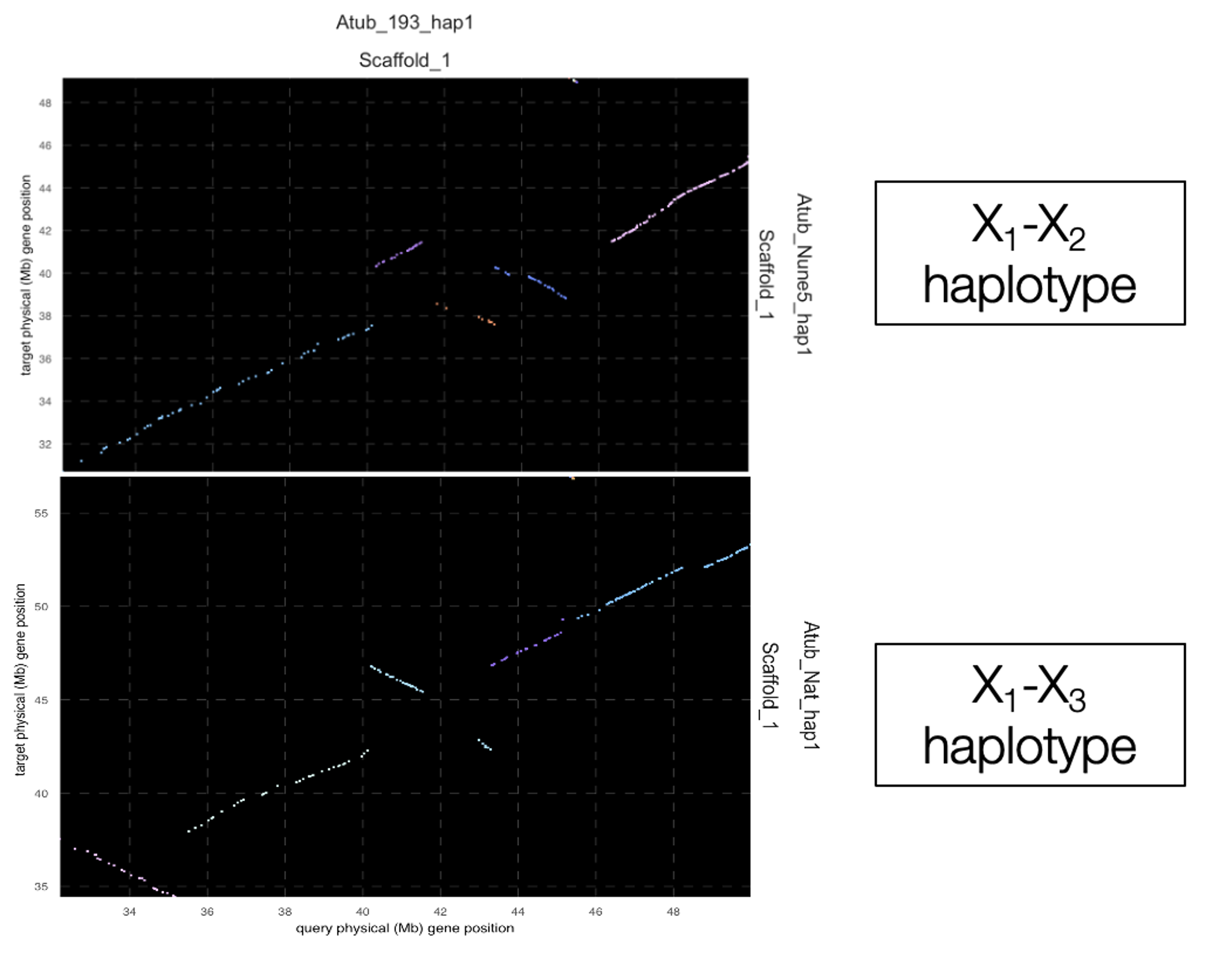

Supplement: S15 Fig — Colors represent orthologous grouping (i.e., tracts of contiguous syntenic sequence). The data underlying this figure can be found in https://zenodo.org/records/15594570. (PNG) [file pbio.3003254.s015.png]

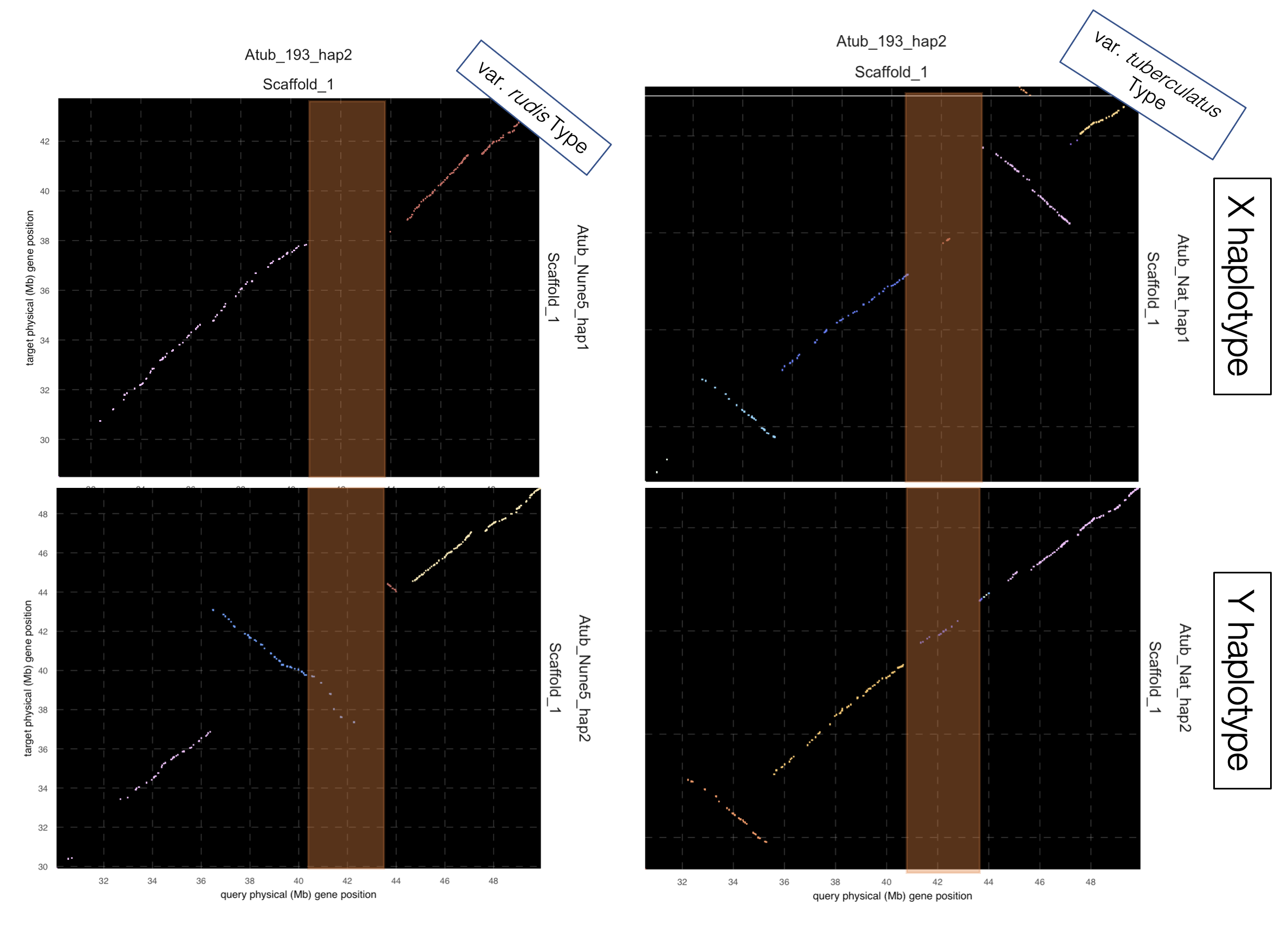

Supplement: S16 Fig — The data underlying this figure can be found in https://zenodo.org/records/15594570. (PNG) [file pbio.3003254.s016.png]

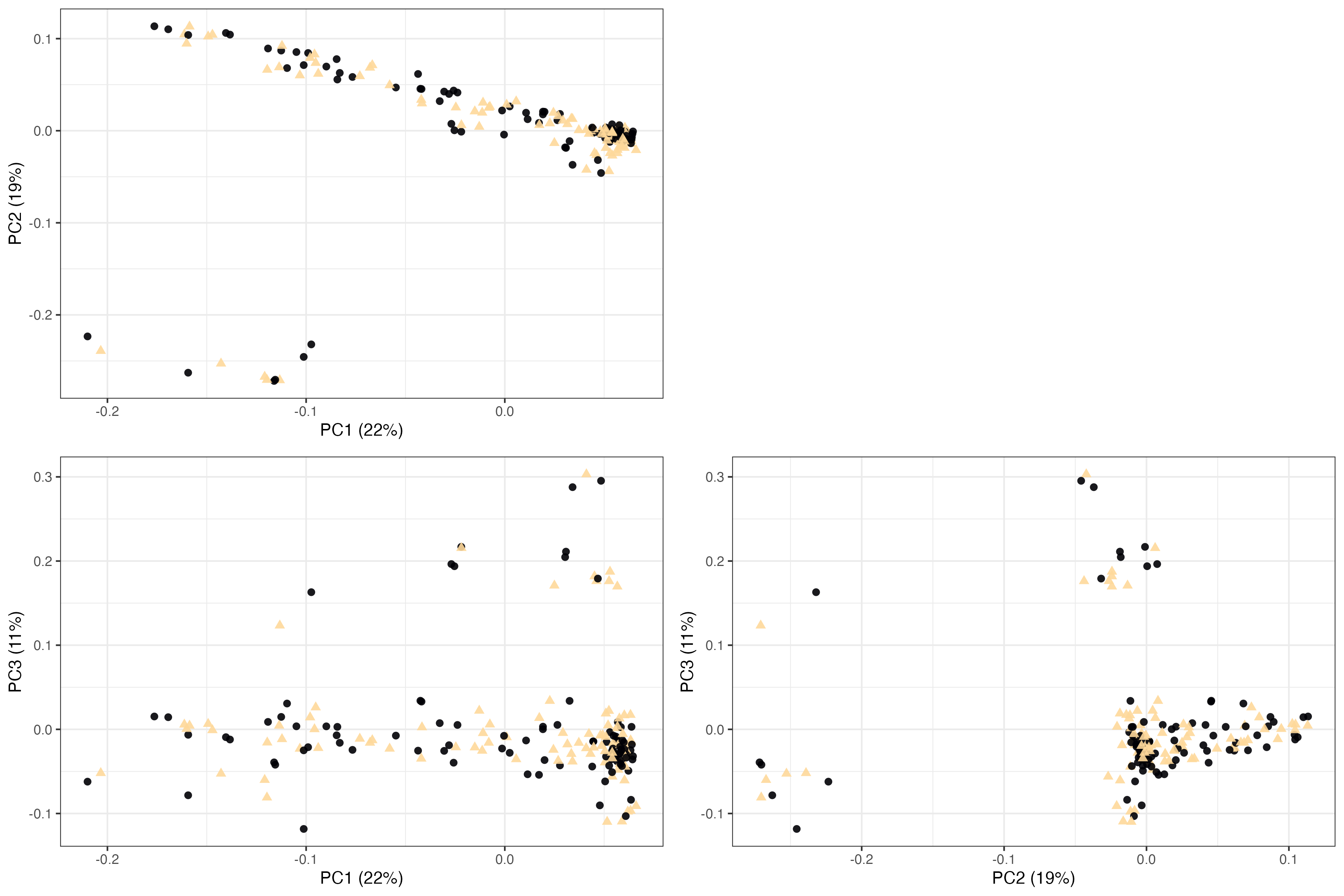

Supplement: S17 Fig — Points (individual resequenced plants) coloured by phenotypic sex assignment (cream = male, black = female), illustrating the lack of sex-based structure in this inversion. The data underlying this figure can be found in https://zenodo.org/records/15594570. (PNG) [file pbio.3003254.s017.png]

Chromosome 1 Sex-Linked Genes

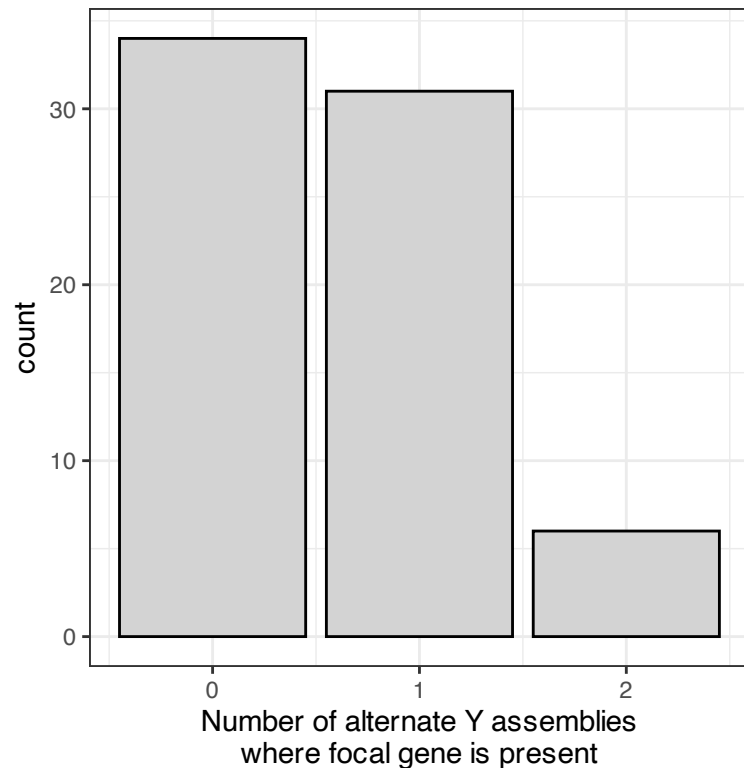

Genome-Wide

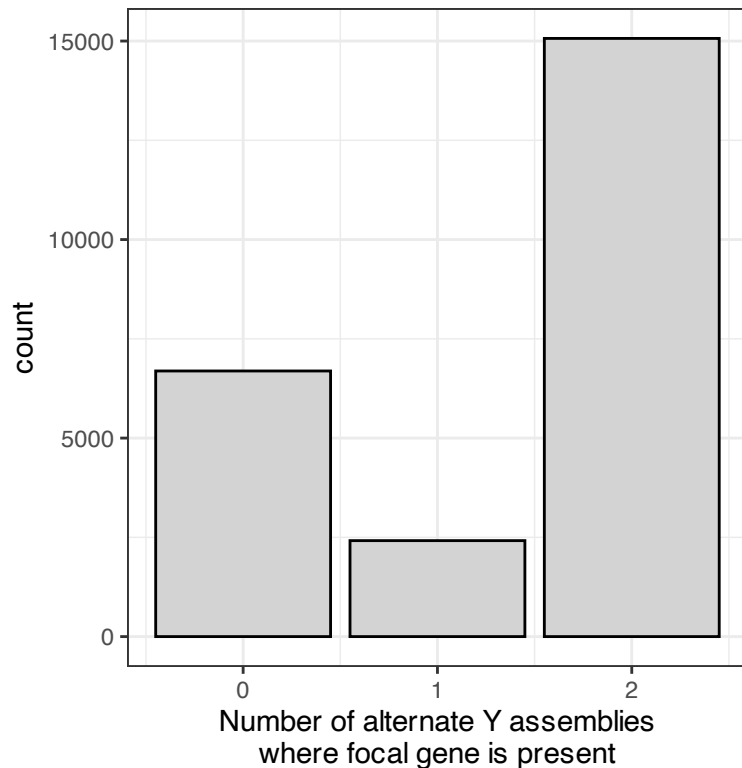

Supplement: S18 Fig — The data underlying this figure can be found in https://zenodo.org/records/15594570. (PDF) [file pbio.3003254.s018.pdf]

Difference in Copy Number  
from Matched-Sex

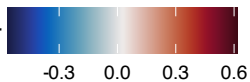

Collection  
Habitat

● Ag ▲ Nat

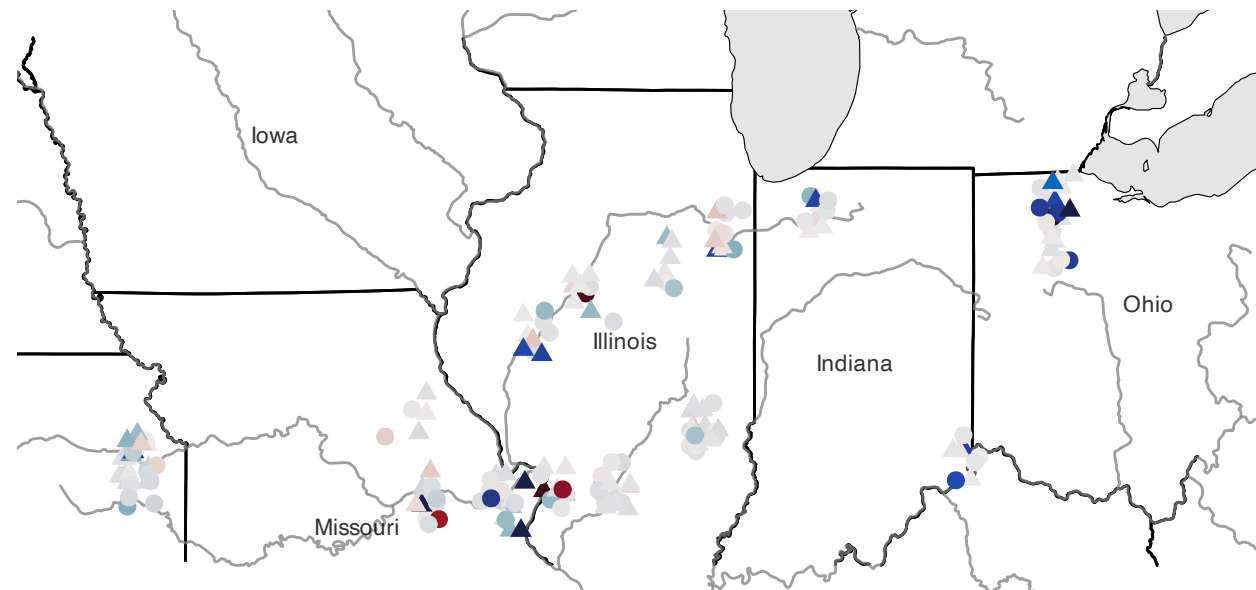

Supplement: S19 Fig — This value repesents the degree of sequenced content mismatch based on an individual’s phenotype. Negative numbers indicate males who genotypically resemble most females, and who therefore have less sequence content in the SLR than most males, whereas positive values indicate females who genotypically resemble most males (having an excess of sequence content in the SLR). Administrative boundaries obtained from GADM (https://gadm.org/) and Natural Earth (https://www.naturalearthdata.com/), while natural features (lakes, rivers, ocean) were obtained exclusively from Natural Earth. Both datasets are freely available for academic use and compatible with CC BY 4.0 licensing. The data underlying this figure can be found in https://zenodo.org/records/15594570. (PDF) [file pbio.3003254.s019.pdf]

Median Scaled Depth in Male Hemizygous Region

Longitude

sex

F  
M

1.5  
1.0  
0.5  
0.0

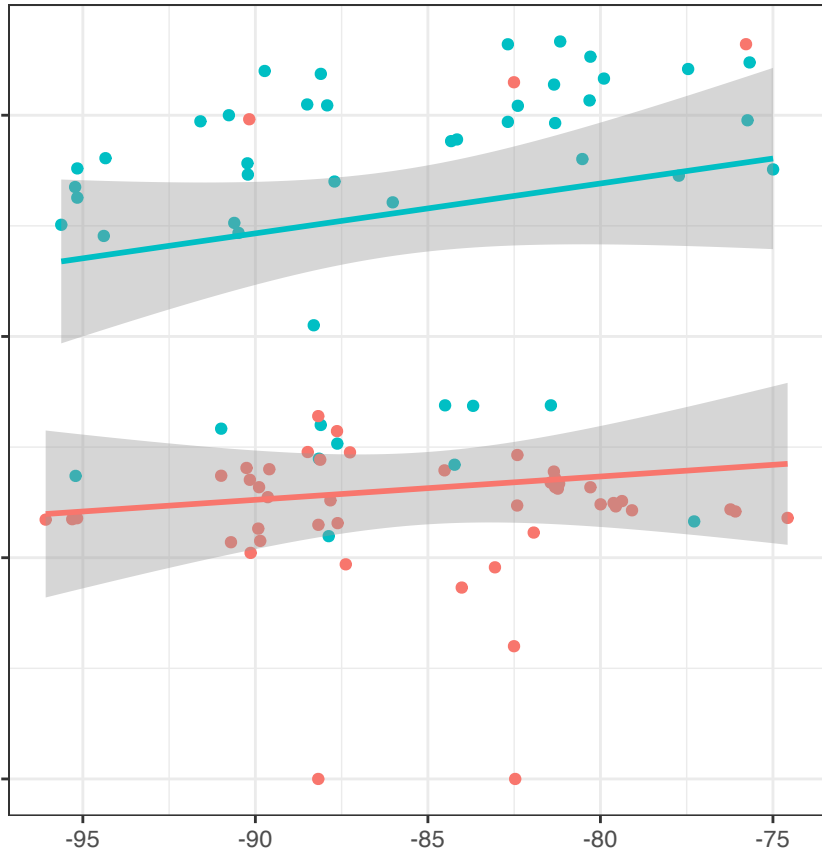

Supplement: S20 Fig — Ten males show depth profiles more similar to females than expected, and three females show depth profiles more similar to females. The data underlying this figure can be found in https://zenodo.org/records/15594570. (PDF) [file pbio.3003254.s020.pdf]

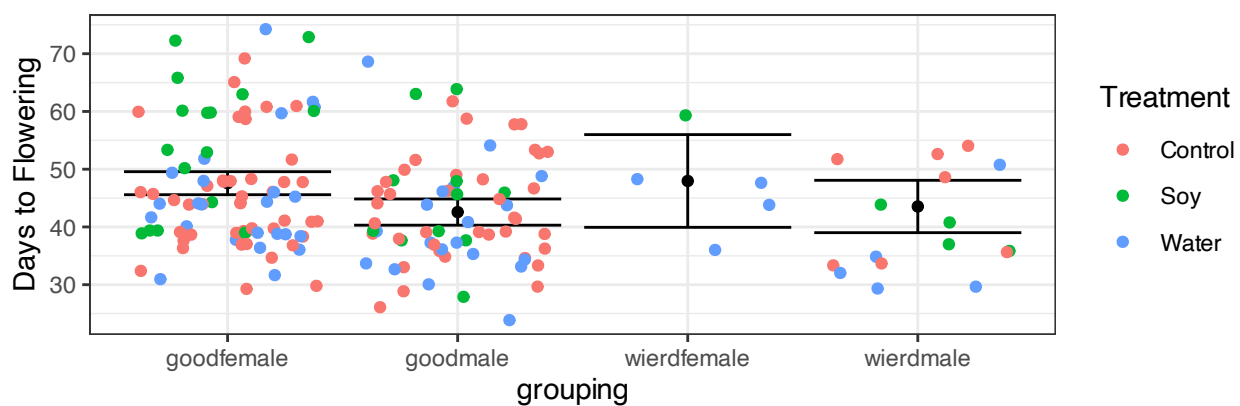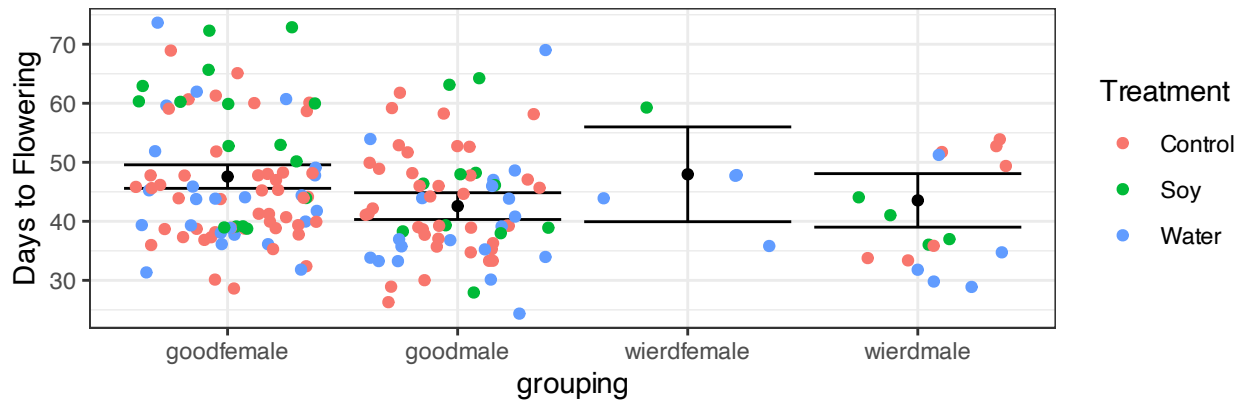

Supplement: S21 Fig — We see no significant difference in phenotypes between mismatched individuals and their respective matched sex. The data underlying this figure can be found in https://zenodo.org/records/15594570. (PDF) [file pbio.3003254.s021.pdf]

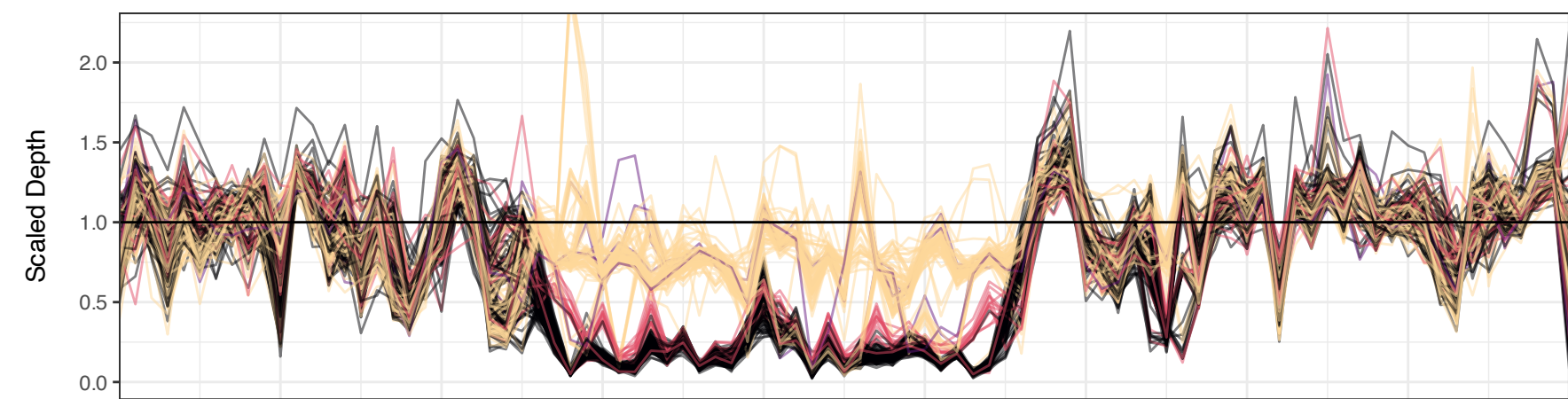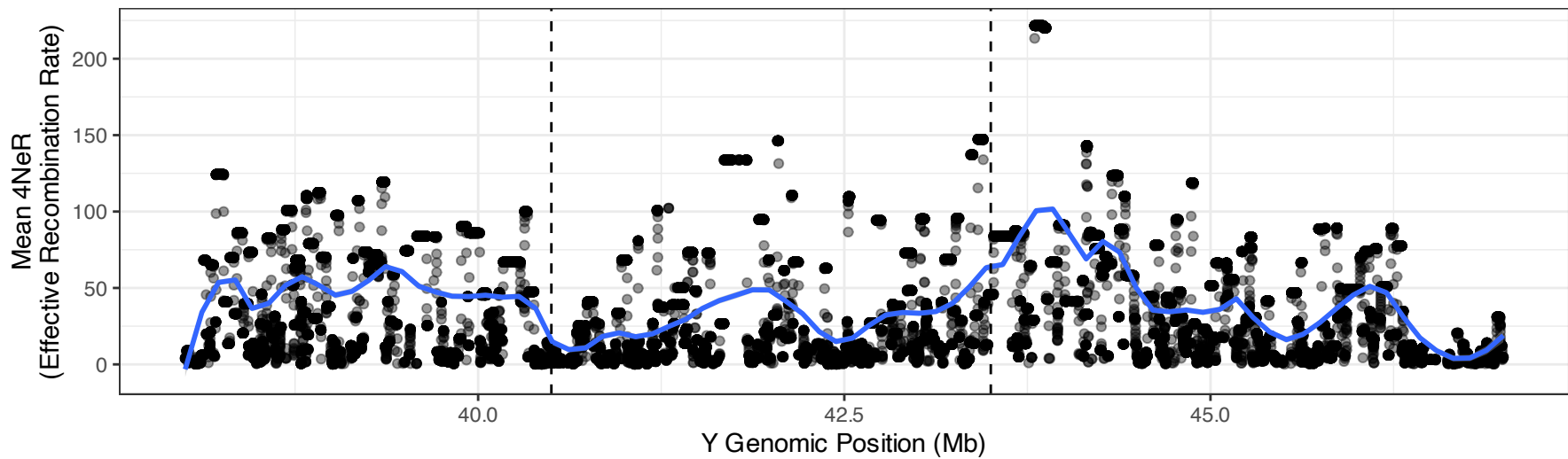

Supplement: S22 Fig — The sex linked region (illustrated by the difference in mapping depth between males and females (top)), shows a reduction in effective recombination rate, as estimated between SNPs along the sex linked region on haplotype 2 (the Y containing haplotype) of Chromosome 1.Vertical black dashed lines delimit the SLR. The data underlying this figure can be found in https://zenodo.org/records/15594570. (PDF) [file pbio.3003254.s022.pdf]

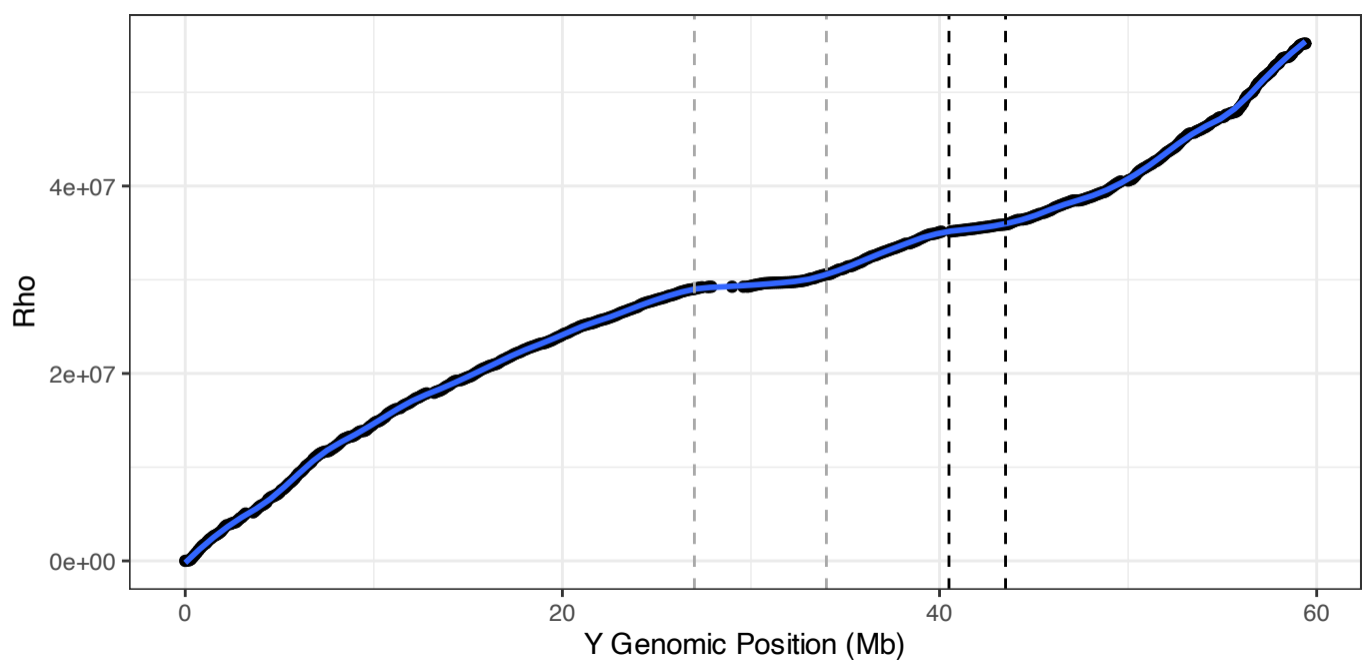

Supplement: S23 Fig — Vertical gray dashed lines represent the centromere, whereas vertical black dashed lines represent the SLR. (PDF) [file pbio.3003254.s023.pdf]
