# Supplementary material for: The evolution of separate sexes in waterhemp is associated with surprising chromosomal diversity and complexity
Source: PLoS Biol. 2025 Jun 30;23(6):e3003254. doi: 10.1371/journal.pbio.3003254 (PMC12237273; doi:10.1371/journal.pbio.3003254)

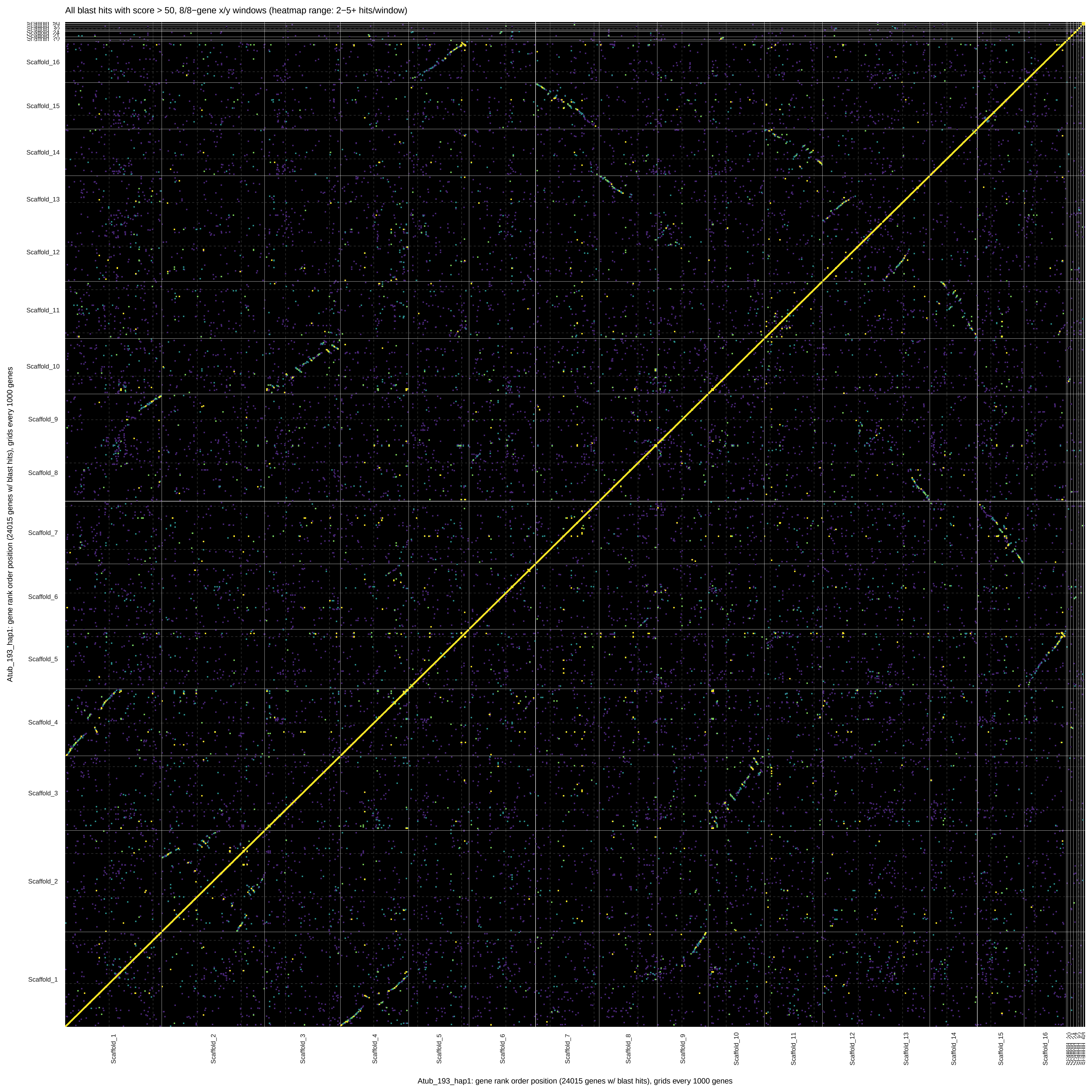

All blast hits with score > 50, 8/8−gene x/y windows (heatmap range: 2−5+ hits/window)
Scaffold_16
Scaffold_15
Scaffold_14
Scaffold_13
Scaffold_12
Scaffold_11
Scaffold_10
Scaffold_9
Scaffold_8
Scaffold_7
Scaffold_6
Scaffold_5
Scaffold_4
Scaffold_3
Scaffold_2
Scaffold_1
Atub_193_hap1: gene rank order position (24015 genes w/ blast hits), grids every 1000 genes
Atub_193_hap1: gene rank order position (24015 genes w/ blast hits), grids every 1000 genes

Blast hits where query and target are in the same orthogroup, 8/8−gene x/y windows (heatmap range: 1−5+ hits/window)

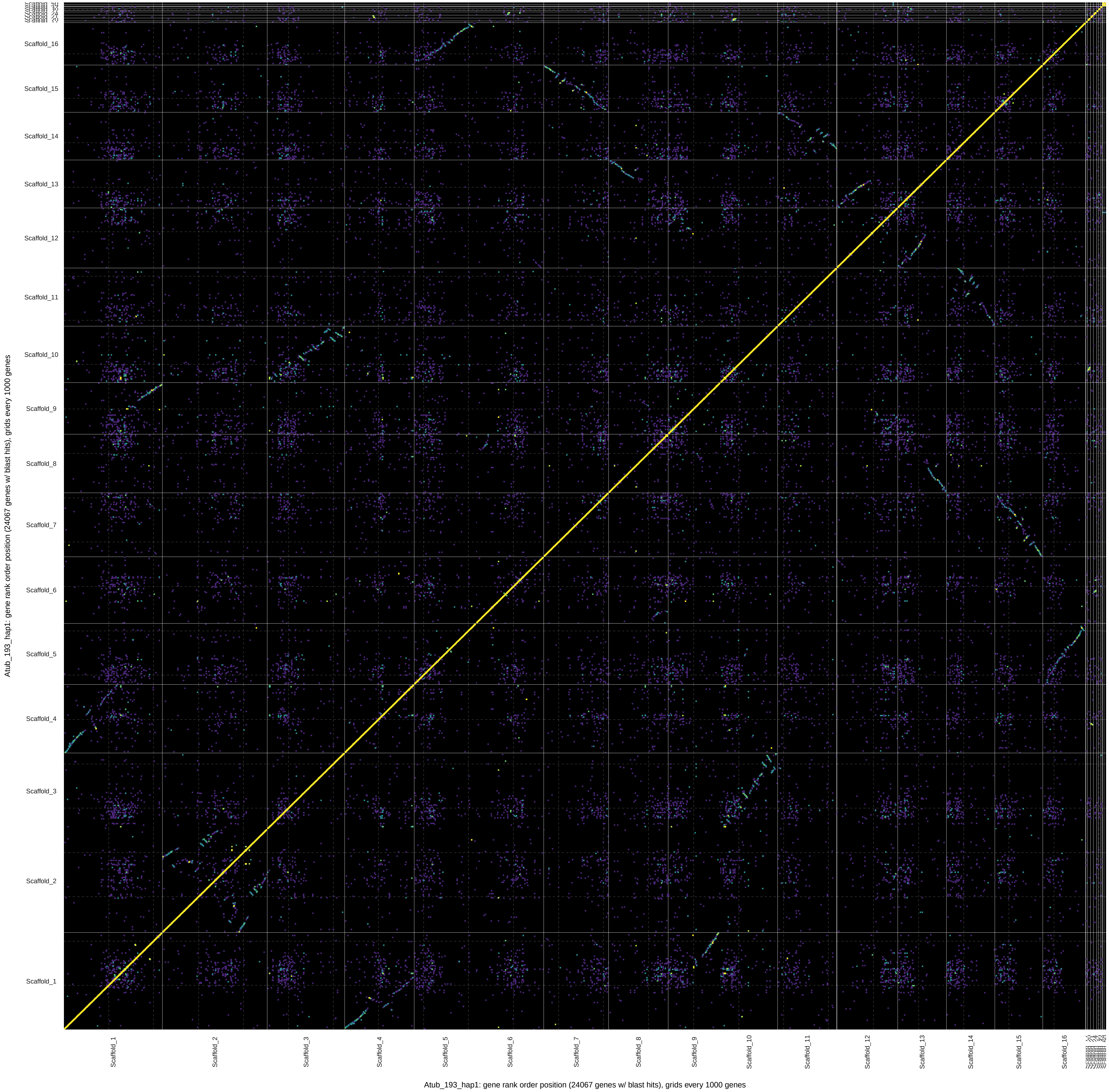

Supplement: S14 Fig — Evidence of a past whole-genome duplication event is apparent as every scaffold shows contiguous stretches of synteny with one or more scaffolds. The data underlying this figure can be found in https://zenodo.org/records/15594570. (PDF) [file pbio.3003254.s014.pdf]
